# Infertility control of transgenic fluorescent zebrafish with targeted mutagenesis of the *dnd1* gene by CRISPR/Cas9 genome editing

**DOI:** 10.3389/fgene.2023.1029200

**Published:** 2023-01-13

**Authors:** Wai-Kwan Chu, Shih-Chin Huang, Ching-Fong Chang, Jen-Leih Wu, Hong-Yi Gong

**Affiliations:** ^1^ Department of Aquaculture, National Taiwan Ocean University, Keelung, Taiwan; ^2^ Center of Excellence for the Oceans, National Taiwan Ocean University, Keelung, Taiwan; ^3^ Institute of Cellular and Organismic Biology, Academia Sinica, Taipei, Taiwan; ^4^ College of Life Sciences, National Taiwan Ocean University, Keelung, Taiwan

**Keywords:** infertility control, fluorescent zebrafish, transgenic fish, genome editing, CRISPR/Cas9, Dead end, PGCs

## Abstract

Transgenic technology and selective breeding have great potential for the genetic breeding in both edible fish and ornamental fish. The development of infertility control technologies in transgenic fish and farmed fish is the critical issue to prevent the gene flow with wild relatives. In this study, we report the genome editing of the *dead end* (*dnd1*) gene in the zebrafish model, using the CRISPR/Cas9 technology to achieve a loss-of-function mutation in both wild-type zebrafish and transgenic fluorescent zebrafish to develop complete infertility control technology of farmed fish and transgenic fish. We effectively performed targeted mutagenesis in the *dnd1* gene of zebrafish with a single gRNA, which resulted in a small deletion (−7 bp) or insertion (+41 bp) in exon 2, leading to a null mutation. Heterozygotes and homozygotes of *dnd1*-knockout zebrafish were both selected by genotyping in the $F_{1}$ and $F_{2}$ generations. Based on a comparison of histological sections of the gonads between wild-type, heterozygous, and homozygous *dnd1* zebrafish mutants, the *dnd1* homozygous mutation (aa) resulted in the loss of germ cells. Still, there was no difference between the wild-type (AA) and *dnd1* heterozygous (Aa) zebrafish. The homozygous *dnd1* mutants of adult zebrafish and transgenic fluorescent zebrafish became all male, which had normal courtship behavior to induce wild-type female zebrafish spawning. However, they both had no sperm to fertilize the spawned eggs from wild-type females. Thus, all the unfertilized eggs died within 10 h. The targeted mutagenesis of the *dnd1* gene using the CRISPR/Cas9 technology is stably heritable by crossing of fertile heterozygous mutants to obtain sterile homozygous mutants. It can be applied in the infertility control of transgenic fluorescent fish and genetically improved farmed fish by selective breeding to promote ecologically responsible aquaculture.

## 1 Introduction

Transgenic technology is a powerful and useful technique to establish transgenic organisms by insertion of a foreign gene into the genome of organisms to obtain or enhance the function of transgene *in vivo* under the control of ubiquitous promoter or tissue-specific promoter (Ozato et al., 1992; Gordon, 1997; Her et al., 2003). Since 1980, scholars have used transgenic technology to improve the performance of major farmed fish species (Du et al., 1992; Maclean, 1993). A transgenic Atlantic salmon, AquAdvantage Salmon, which expressing growth hormone of Chinook salmon was established under the control of a promoter from ocean pout and obtained the approval for consumption in the U.S. on November 2015 and 6-month later in Canada. AquAdvantage Salmon grows twice faster to market size than non-transgenic Atlantic salmon counterpart. Thus, transgenic technology has great potential for the development of aquaculture, as it significantly reduces farming costs and risks (Cook et al., 2000; Muir, 2004). With the subsequent simplification of the transgenic procedures and methods, the application of transgenic technology gradually became popular (Kawakami, 2007; Suster et al., 2009). Besides in the food fish aquaculture industry, transgenic technology has been applied in the ornamental industry (Gong et al., 2003). Fluorescent fish is a concept originated by scholars using the transgenic technique to visualize and calibrate tissues to study specific gene regulatory mechanisms and functions (Ju et al., 1999; Gong et al., 2001; Chudakov et al., 2010). According to the ornamental fish industry data, fluorescent fish are new varieties that meet consumers’ demands in terms of unique body colors, posing a huge potential business opportunity, which may enable the industry to become more competitive (Stewart, 2006). Transgenic fish applications are expected to increase exponentially in both the aquatic and ornamental fish industries globally. If these transgenic fish escape and interbreed with wild stock, this poses a potential threat to the ecosystem and environment (Hu et al., 2007). Recently, transgenic glowing zebrafish appear to be thriving after escape from fish farms into Brazilian streams and may threaten local biodiversity (Magalhaes et al., 2022; Moutinho, 2022). We believe that developing practical infertility control technology is the most effective way to avoid ecological risks, and it also promotes the development of an environmentally responsible aquaculture (Muir and Howard, 1999; Stigebrandt et al., 2004; Wong and Van Eenennaam, 2008).

The method of establishing infertile individuals has been continuously developed. The methods commonly used to produce infertile fish are triploidization and interspecies hybridization (Benfey et al., 1989; Arai, 2001; Cal et al., 2006); however, some treated individuals were found to maintain fertility and show limitations, depending on the species (Wagner et al., 2006; Piferrer et al., 2009). In our previous research, we tried to use the transgenic strategy, transferring the germ cells’ specific gene *piwi* promoter to be combined with the nitroreductase toxic protein (NTR). When the metronidazole (Mtz) bath immersion treatment succeeded, cell apoptosis began due to the conversion of Mtz into a cytotoxic compound, which may affect the germ cells and induce gonadal development disruptions. However, the effects of the NTR/Mtz system are not obvious. While the methods mentioned above failed, the disruption of the mechanisms of primordial germ cell formation and migration was successful in achieving complete infertility (Wong and Collodi, 2013; Wong and Zohar, 2015a; Wong and Zohar, 2015b).

Primordial germ cells (PGCs) are the progenitor cells of gametes. PGCs play an important role, as egg and sperm production relies on the formation, differentiation, and correct localization of PGCs (Yoon et al., 1997; Weidinger et al., 1999; Xu et al., 2010; Yamaha et al., 2010). In zebrafish, the development of PGCs has been studied extensively. PGC formation occurs during early embryogenesis maternally through a germplasm composed of maternal RNA-binding proteins and mRNAs (Braat et al., 1999; Extavour and Akam, 2003; Raz, 2003). In addition, the germ cell marker *ddx4* also named as *vasa*, an evolutionarily conserved gene, was first discovered to label PGCs successfully, and it was shown to be a key factor in the migration of PGCs to the genital ridge (Yoon et al., 1997; Braat et al., 1999; Castrillon et al., 2000; Yoshizaki et al., 2000). However, by inhibiting the gene expression by gene silencing in different species, it did not induce a state of a complete loss of germ cells or infertility, resulting in a single sex (Raz, 2000; Li et al., 2009; Hartung et al., 2014). There is no evidence proving that no other cells can potentially contribute to the germline during normal development or when the number of *ddx4*-expressing cells is reduced. The *dead end (dnd1)* gene, encoding an RNA-binding protein DND1, was first identified in zebrafish (Weidinger et al., 2003) and is highly conserved in vertebrate species (Horvay et al., 2006; Aramaki et al., 2007; Liu et al., 2009; Nagasawa et al., 2013). The expression of some germline-specific mRNAs relied on the protection of DND1 from microRNA-mediated inhibition (Kedde et al., 2007a). The DND1 knockdown by translational inhibition of maternal *dnd1* mRNA with Morpholino antisense oligonucleotides resulted in PGCs losing their ability to migrate actively and mis-migration, followed by apoptosis or transdifferentiation into somatic cells in zebrafish (Weidinger et al., 2003; Gross-Thebing et al., 2017). The *dead end* (*dnd1*) knockout fish by genome editing was first conducted in medaka with TALENs and showed germ cell-less gonads (Wang and Hong, 2014; Wang, 2015). The *dnd1* knockout zebrafish was first established by genome editing with Zinc Finger Nucleases (ZFNs) as germ cells-less recipients of surrogate for germ cell transplantation (Li et al., 2017). However, compared to ZFN and TALEN, which rely on DNA binding domain to recognize DNA, the CRISPR/Cas9 system by using a single guide RNA (sgRNA) for DNA recognition is more efficient, convenient, and cost‐effective (Doudna and Charpentier, 2014). Therefore, CRISPR/Cas9 technology had been applied in genome editing of several fish species such as Atlantic salmon, medaka, sterlet, and rainbow trout to target *dnd1* to achieve infertility (Wargelius et al., 2016; Sawamura et al., 2017; Baloch et al., 2019; Baloch et al., 2021; Fujihara et al., 2022).

CRISPR/Cas9 technology has been widely used due to its ease of operation, which allows scholars to investigate gene function (Ran et al., 2013). Genome editing used the Cas9 protein and guide RNA (gRNA), and the 5’ of gRNA contained an 18–20 bp protospacer sequence that could identify the target sequence and guide Cas9 to cause a DNA double-strand break, thus inducing the mechanism of DNA repair, i.e., non-homologous end joining (NHEJ) and homology-directed repair (HDR) (Garneau et al., 2010; Horvath and Barrangou, 2010; Hwang et al., 2013). There is an opportunity to cause an insertion or deletion through the NHEJ repair mechanism, and an in-frame stop codon may emerge through the insertion or deletion performed by CRISPR/Cas9, causing the translation to stop early, which results in a loss of gene function due to early truncation of the protein (Chapman et al., 2012; Auer et al., 2014).

While transgenic technology has a huge potential to be applied in aquaculture, it poses unexpected risks to the ecosystem, as the organisms maintain the ability to crossbreed with wild stock. Developing effective and practical infertility control is the key to solving the problem. Although *dnd1* knockout in zebrafish was achieved by ZFNs (Li et al., 2017), CRISPR/Cas9 technology was still not applied in *dnd1* knockout of zebrafish, especially in new application of infertility control of transgenic fluorescent zebrafish as a model of fluorescent ornamental fish. The phenotypes of homozygous and heterozygous *dnd1* knockout zebrafish by CRISPR/Cas like those by ZFNs are not novel but convince us of no off-targeting effect in *dnd1* knockout zebrafish by both genome editing technologies. In this study, we demonstrate that the *dnd1* knockout by CRISPR/Cas9 genome editing had been successfully applied to the infertility control of transgenic fluorescent zebrafish as a model of transgenic fluorescent ornamental fish to prevent potential impact on ecology when they escape into the wild field.

## 2 Materials and methods

### 2.1 Establishment of *dnd1*-knockout zebrafish using the CRISPR/Cas9 system

Wild-type (AB) strain zebrafish were used as experimental animals in this study. The zebrafish were maintained in a recirculating system, with the temperature maintained at 28°C and photoperiodism of 14 h of light and 10 h of dark. The animal use protocol of this research had been reviewed and approved by the Institutional Animal Care and Use Committee (IACUC) of National Taiwan Ocean University. The IACUC Approval No. is 108042. The CRISPR/Cas9 system was used to establish the *dnd1*-knockout zebrafish line (Baloch et al., 2021). The sgRNA target site was designed using CHOPCHOP, using an off-target assay for analysis (Labun et al., 2016). The pCS2-nCas9n was a gift from Wenbiao Chen (Addgene plasmid # 47929; http://n2t.net/addgene:47929; RRID: Addgene_47929) for Cas9 mRNA synthesis by *in vitro* transcription (Jao et al., 2013). The zebrafish *dnd1* target-specific DNA oligo (5′-TTC​TAA​TAC​GAC​TCA​CTA​TAGTAA​CCC​AAG​TCA​ATG​GGC​AGGTT​TTA​GAG​CTA​GA-3′) was synthesized by “Genomics” company (Taiwan) for sgRNA synthesis. The sgRNA was synthesized based on the protocol of EnGen^®^ sgRNA synthesis kit (New England Biolabs, United States). The zebrafish reproduction was performed under the conditions mentioned above. The embryos were collected to perform the microinjection. The microinjection was performed by co-injecting a mixture of reagents—300 ng/μL of Cas9 mRNA and 30 ng/μL of sgRNA—into one-cell-stage embryos. The injected embryos were cultivated in a Petri dish at 28°C for hatching. To predict the efficiency of the gRNA targeting, 10 individuals were randomly selected 24 h after fertilizing (hpf) the injected embryos for DNA extraction, and the T7 endonuclease I (T7E1) assay was used to evaluate the efficiency of the gRNA target site mutation (Urnov et al., 2005).

### 2.2 Mutation screening by sequencing in second-generation ( $F_{1}$ ) zebrafish

The $F_{1}$ generation was produced by crossing the first-generation ( $F_{0}$ ) with wild-type and inbreeding of the first-generation ( $F_{0}$ ). Genomic DNA from the caudal fin of each $F_{1\;}$ fish larva was extracted to analyze the genotype (Meeker et al., 2007). A 150 bp fragment of the target site region was amplified using the 5× PCR Dye Hot Start Master Mix (GeneMark, Taiwan), using the following primers: 5′-ATT​CTG​AAC​CCG​CAG​AAA​CTC​AAG​TCT​CTG​CAG​GA and 3′-AAT​GCT​GCT​CTC​AGG​TCG​ATG​GAG​GGG​CAC​TTA​C. The genotype analysis was performed by gel electrophoresis using a 2% agarose gel, followed by the gene cloning of the individuals to confirm the mutations. The sequences were analyzed using the Molecular Evolutionary Genetics Analysis Version 7.0 (Mega 7) software to predict the form of protein (Kumar et al., 2016; Bhattacharya and Van Meir, 2019). Heterozygotes whose mutations were predicted to show a stop codon were selected to develop the $F_{1\;}$ generation. The same mutation pattern of heterozygotes in the $F_{1\;}$ generation was inbred to establish the $F_{2}$ generation. The genotyping process mentioned above was performed to interpret the *dnd1-*knockout heterozygotes and homozygotes, and the number of individuals of different genotypes was recorded to calculate the heritability.

### 2.3 Establishment of the fluorescent *dnd1*-knockout zebrafish line

The transgenic fluorescent zebrafish line *Tg(-2.4ckmb:TcCFP13)*, expressing the Taiwan coral (*Acropora* sp.) cyan fluorescent protein, TcCFP-13 cDNA (provided by Dr. Ming-Chyuan Chen, National Kaohsiung University of Science and Technology, Taiwan), driven by a novel zebrafish muscle-specific *ckmb* 2.4 kb promoter/enhancer (GenBank accession number HM347596), was previously established *via* the *Tol2* transposon system (Gong et al., 2015). The fluorescent zebrafish strain was mated with the *dnd1*-knockout heterozygotes to establish the $F_{1}$ generation fluorescent *dnd1*-knockout fish line. The individuals with cyan fluorescent embryos were selected after 72 hpf, followed by the genotype analysis process mentioned above to select the heterozygotes. The cyan fluorescent *dnd1*-knockout homozygous zebrafish were obtained by inbreeding with the F_1_ *dnd1*-knockout heterozygotes.

### 2.4 PGC localization analysis by whole-mount *in situ* hybridization

The sense and antisense *ddx4* riboprobes were designed in the open reading frame of zebrafish *ddx4*, and the primers of the probe were 5′-GCG​TGT​CCA​CCT​GCT​ACC​GGC​TCT​TCT​GAA and 3′-TTC​ATC​ACG​GGA​GCC​ACT​GCG​AAA​ACC. The riboprobes were synthesized using the DIG RNA Labeling Kit (SP6/T7) (Roche) from linearized pGEM-T *ddx4.* The embryos were collected 24 h post-fertilization, followed by fixation with 4% paraformaldehyde. The signals were detected by NBT/BCIP staining (Brend and Holley, 2009). We selected and separated the embryos with a positive and negative signal under a Leica EZ4 microscope (Leica, Germany), after capturing the phenotypes. Then, the embryos were washed 5 times using 1× PBS, followed by DNA extraction with the MasterPure™ DNA Purification Kit (Epicentre, United States). PCR amplification was performed using the 5× PCR Dye Hot Start Master Mix (GeneMark, Taiwan). The genotype was analyzed by gel electrophoresis using a 2% agarose gel.

### 2.5 Histological analysis of zebrafish gonadal tissue

Three individuals from the wild types, heterozygotes, and homozygotes in the female and male groups were selected. The sample (whole fish) was fixed with Davidson’s fixation solution for 24 h; then, the sample was kept in 70% ethanol (Miki et al., 2018). The sample was dehydrated with ethanol and then embedded into paraffin blocks. Then, the sample was cut into 5 μm-thick sections, and the sections were mounted on glass slides in a 42°C thermostatic water bath. Staining was performed using hematoxylin and eosin (HE stains), and the sections were analyzed using an optical microscope (Blazer, 2002).

### 2.6 Semi-quantitative PCR analysis of the gene expression level

Three individuals from the wild types, heterozygotes in the female and male groups, and homozygotes were selected for sampling of the gonadal tissues. The total RNA of the gonads was extracted using Ambion™ TRIzol Reagent and purified with the PureLink™ RNA mini kit. Reverse transcription was performed to synthesize the corresponding cDNA. The primers used in this study were as follows: *ddx4* (forward: 5′-ATG​GAT​GAC​TGG​GAG​GAA​GAT​CAG​AGT​CCC​G-3’; reverse: 5′- TTC​CAT​TTT​CAT​CAT​TTT​CAT​CAC​GGG​A-3′); *dazl* (forward: 5′-ATGGTTCAGGGGG TTCAGTTACCCGTGT-3’; reverse 5′-TGA​TGG​TGG​GGC​CAG​GCC​TGG​AGG​ACA​GCA-3′); *nanos3* (forward: 5′-TTC​TGG​AAT​GAC​TAT​CTC​GGC​CTG​TCC​A-3’; reverse: 5′-ATT​GTG​TGC​GCG​TTG​TCC​CCG​TTG​GCA​CTG​C-3′); *tdrd7a*: (forward: 5′-ATG​GCG​GAC​GAG​GAA​CTG​GTG​AAG​AA-3’; reverse: 5′-CCG​CGA​CCT​CCA​CCT​CGC​CCC​AGA​CGT​CCA​C GG-3′); *amh* (forward: 5′-ATG​CTT​TTC​CAG​GCA​AGA​TTT​GGG​CTG​ATG-3’; reverse 5′- ACT​GTC​TCC​TTT​AAC​AGG​ATT​GAC​ACT​GAA​CAG-3′); and *eef1α1l1* (forward: 5′-TGCCTTCG TCCCAATTTCAG-3’; reverse: 5′-CTC​ATG​TCA​CGC​ACA​CAG​CAA​AG-3′). Semi-quantitative PCR was performed using the following procedure: 94°C for 2 min; 94°C for 30 s; 60°C for 30 s; 72°C for 1 min per kb (repeated for 25–35 cycles); 72°C for 5 min; the reaction was stopped at 4°C. The semi-quantitative PCR analysis was performed by gel electrophoresis using a 1% agarose gel.

### 2.7 Courtship behavior analysis of *dnd1*-knockout zebrafish

The *dnd1-*knockout homozygotic individuals were selected and crossbred with wild-type female zebrafish randomly. The wild-type groups were selected randomly as a control. The photoperiodism was 14 h light and 10 h dark, and the observation was performed during breeding, collecting the eggs of every group and counting and recording photos every hour. The same process was used for the cyan fluorescent *dnd1-*knockout homozygotic groups.

## 3 Results

### 3.1 Establishment of the *dnd1*-knockout zebrafish line

#### 3.1.1 Targeted mutagenesis of the *dnd1* gene in zebrafish using CRISPR/Cas9

**FIGURE 1:**
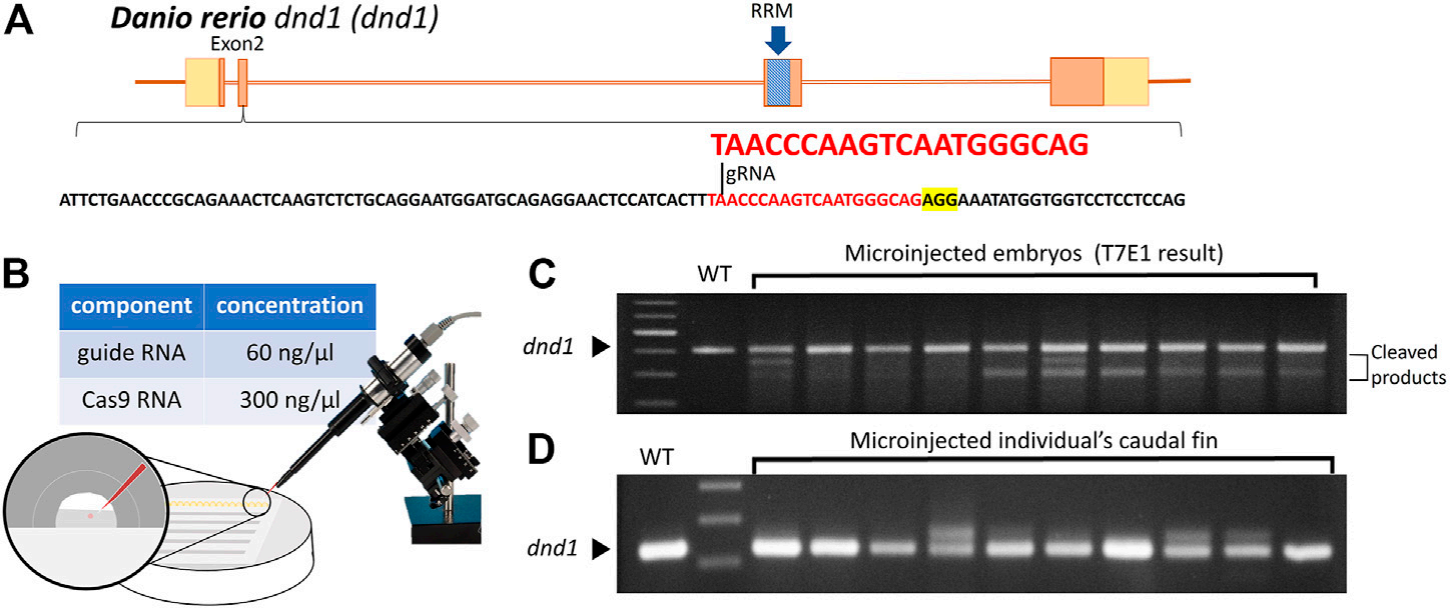
**(A)** Design of the CRISPR/Cas9 target sites of the guide RNA (gRNA) in *dnd1*. The gRNA was designed in exon 2, and the target site of the gRNA is indicated in red, while the sequence highlighted in yellow is PAM. The RNA-recognition motif (RRM) is the conserved functional domain encoded by exon 3 of the *dnd1* gene. **(B)** Microinjection schematic with the component and concentration of the working solution. **(C)** Mutation identified in each $F_{0}$ generation by T7E1 using 2% agarose gel. **(D)** Mutation efficiency analyzed using PCR on the caudal fin’s DNA.

According to previous studies on the *dnd1* gene in zebrafish, the translation region that starts to form the key functional domain, the RNA-recognition motif (RRM), is located in exon 3. The target site of a single gRNA was designed to locate in the second exon of the *dnd1* gene in zebrafish to disrupt the formation of the RNA-recognition motif (Figure 1A). The components of synthesized gRNA and Cas9 mRNA were injected into one cell of fertilized zebrafish embryos (Figure 1B). The injected embryos were cultivated in Petri dishes, and at 24 h post-fertilization (hpf), we randomly selected ten embryos to investigate the efficiency of the target site mutation generated by gRNA using the T7E1 assay. According to the T7E1 results, in nine out of 10 embryos, multiple banding patterns were observed, with some individuals showing significant second major band patterns, revealing DNA mismatches formed in a fraction of heteroduplexes and cleaved by T7E1 (Figure 1C). It was confirmed that the mutation was constituted through the gRNA, and *dnd1* may be successfully disrupted by the CRISPR/Cas9 genome editing system in zebrafish. The $F_{0}$ generation was maintained until sexual maturity; then, the knockout effect was evaluated by extracting the DNA from the caudal fin, followed by PCR and gel electrophoresis to select the individuals. We predict a higher genetic efficiency for heritable mutations, which appeared in multiple bands (Figure 1D). Next, we conducted the inbreeding of selected F0 individuals to establish the $F_{1}$ generation.

#### 3.1.2 Inheritable mutation analysis of $F_{1}$ generation

**FIGURE 2:**
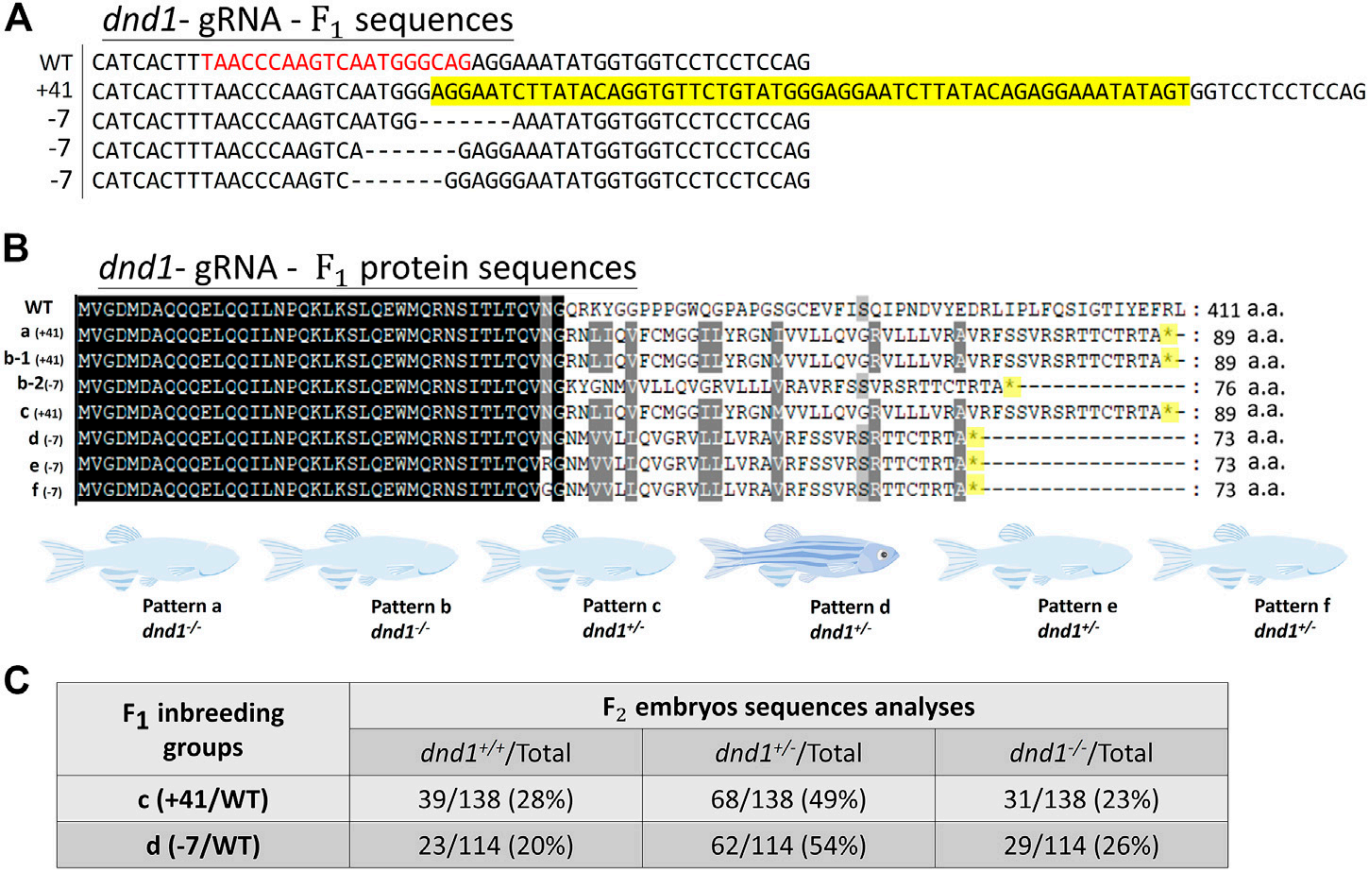
**(A)** Mutation sequences identified in the $F_{1}$ generation by sequencing. The analyzed individuals are shown in order of the mutation sequence size of the insertions (+)/deletions (−). The target site of gRNA is indicated by red characters, and the yellow highlighted sequences are insertional sequences. **(B)** Mutation amino acid sequences identified in each $F_{1}$ zebrafish by genotyping. The in-frame stop codon is shown as * and highlighted in yellow. **(C)** Analysis of the ratio of different genotypes in $F_{2}$ generation.

We further analyzed the mutation patterns of the $F_{1}$ generation. The fish larvae were cultivated for 1 month, and the caudal fin was cleaved for DNA extraction, following by PCR amplification for every individual. In comparing the bands of the PCR product of the wild-type, the individuals with the extra band were separated. To investigate whether the unexpected insertion or deletion mutation patterns in the coding sequence could generate an in-frame stop codon, we performed cloning and used the MEGA 7 software to conduct sequencing. In the $F_{1}$ generation, a total of 120 $F_{1}$ individuals were screened and analyzed. We observed several mutation patterns, five of which had effects on the coding sequence, and the obtained sequence patterns were deletions of seven base pairs (bp) in different regions and insertions of 41 bp (Figure 2A). As the patterns mentioned above resulted in an in-frame shift of the coding sequence, the stop codon was predicted to appear in the protein translation process. We classified the $F_{1}$ zebrafish mutation patterns as a, b, c, d, e, and f. In total, 10 out of 120 individuals were heterozygotes (c, d, e, and f), and two were homozygotes. Besides the heterozygotes, we only obtained two homozygous mutant F_1_ individuals in the a and b patterns by inbreeding the F_0_ generation. Considering the genetic background of F_1_ generation may be affected by the inbreeding of F_0_, we crossed the F0 zebrafish with wild-type to generate the c, d, e, and f strains to eliminate the noise of genetic background for this study. Pattern b, in particular, shows two different mutant alleles (b-1: +41 bp and b-2: −7 bp) (Figure 2B). To generate the stable heritage of the *dnd1-*knockout zebrafish fish line, subsequent mating was performed using individuals identified as heterozygotes patterns c and d (as d, e, and f’s predicted amino acid sequences were identical). In the $F_{2}$ generation, three genotypes were obtained: wild-type (AA, ${\mathrm{dnd}1}^{+/+}$ ), heterozygotes (Aa, ${\mathrm{dnd}1}^{+/-}$ ), and homozygotes with a mutation in a pair of alleles (aa, ${\mathrm{dnd}1}^{-/-}$ ). To investigate the preliminary data of heterozygotes and homozygotes that might have been produced after inbreeding, two groups of $F_{2}$ offspring were subjected to genotyping 30 days post-fertilization. The results for the genotypes’ heritability ratios were similar for the two groups. In the mutation pattern c fish line, among the total of 138 individuals that we subjected to genotyping, 68 individuals (49%) were found to be ${\mathrm{dnd}1}^{+/-}$ (Aa), which is the most populous group, 39 individuals (28%) were found to be ${\mathrm{dnd}1}^{+/+}$ (AA), and 31 individuals (23%) were found to be ${\mathrm{dnd}1}^{-/-}$ (aa) (Figure 2C). In the mutation pattern d fish line, 62 individuals (54%) found to be ${\mathrm{dnd}1}^{+/-}$ (Aa) accounted for the highest proportion of the population among 114 individuals, 29 individuals (26%) were found to be ${\mathrm{dnd}1}^{-/-}$ (aa), and 23 individuals (20%) were found to be ${\mathrm{dnd}1}^{+/+}$ (AA) (Figure 2C). Considering the definiteness of the following experiments, we decided to further analyze the stain of pattern D with a heritable 7 bp deletion.

### 3.2 PGC and gonad analyses of *dnd1* zebrafish mutants

#### 3.2.1 PGC localization analysis of the *dnd1*-knockout zebrafish larva

**FIGURE 3:**
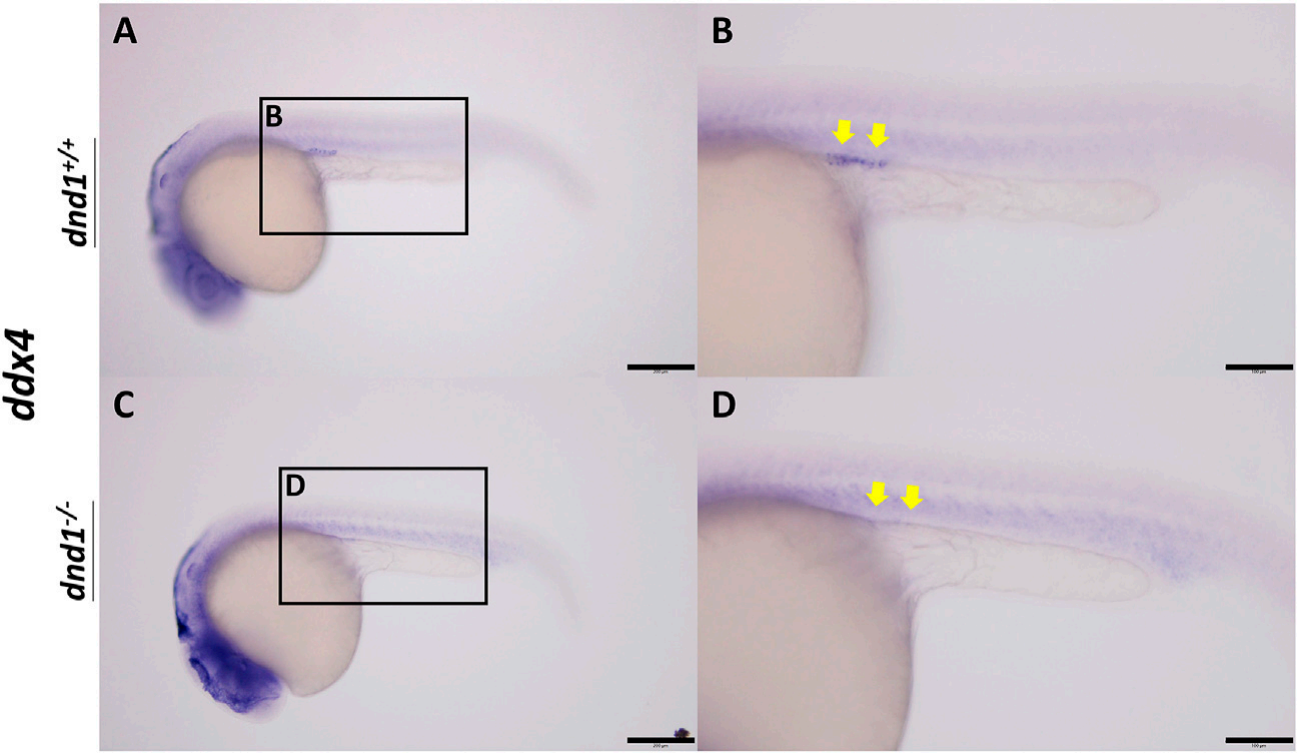
*ddx4* expression visualized by whole-mount *in situ* hybridization in 24-h post-fertilized embryos of the $F_{2}$ generation. **(A)**-**(B)** Wild-type zebrafish embryo. **(C)**-**(D)** Mutant zebrafish embryo. Bars = 200 μm **(A, C)** and 100 μm **(B, D)**.

In wild-type zebrafish, PGCs may be observed by whole-mount *in situ* hybridization in 24 hpf embryos localized on the genital ridge. We performed the inbreeding of pattern D heterozygotes to collect the fertilized embryos. To investigate whether the PGCs localized normally on the genital ridge in *dnd1*-knockout offspring, the germ cell marker *ddx4* was used as an RNA probe label for PGC visualization. In the ${\mathrm{dnd}1}^{+/+}$ (AA) individuals, we observed cell-like positive signals in dotted particles on the lateral view, localized in position straight in front of the tube-like region of the embryos (Figures 3A, B). Conversely, no positive-signal cells were found to be localized in the same position as the ${\mathrm{dnd}1}^{-/-}$ (aa) embryos (Figures 3C, D). As *ddx4* is specifically expressed in PGCs, starting from the earliest stage of embryonic development, a loss of *ddx4* signal in mutant individuals represents loss of PGCs. Furthermore, we focused on the development of gonads in sexually mature individuals to discover whether *dnd1* knockout led to infertility.

#### 3.2.2 Gonadal tissue analysis of the mature *dnd1*-knockout zebrafish

**FIGURE 4:**
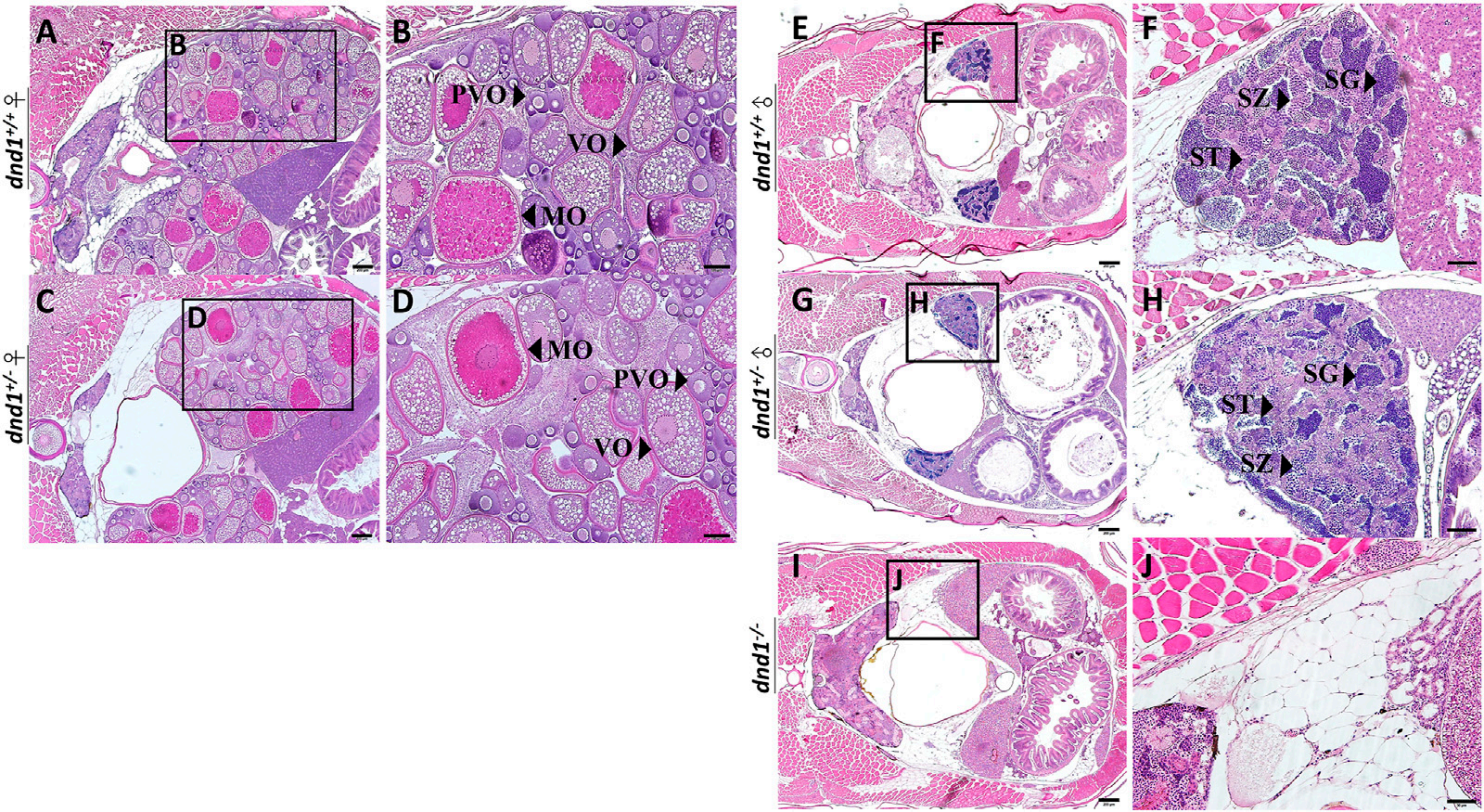
Histological analyses of zebrafish gonad. **(A)**-**(B)** Wild-type zebrafish ovary. **(C)**-**(D)** Heterozygote zebrafish ovary. **(E)**-**(F)** Wild-type zebrafish testis. **(G)**-**(H)** Heterozygote zebrafish testis. **(I)-(J)** Mutant zebrafish testis. PVO: previtellogenic oocytes; VO: vitellogenic oocytes; MO: mature oocyte; SC: spermatocytes; SG: spermatogonia; ST: spermatids. Bars = 200 μm **(A, C, E, G, I)**, 100 μm **(B, D)** and 50 μm **(F, H, J)**.

We separated the individuals according to the three groups of genotypes and randomly selected both male and female individuals from each genotype. No individual with the female phenotype was obtained from the ${\mathrm{dnd}1}^{-/-}$ (aa) groups. Histological analysis was performed by tissue sectioning, and the tissue was stained with hematoxylin and eosin (HE staining). In the ${\mathrm{dnd}1}^{+/+}$ (AA) female individuals, we observed a completely developed ovary, with primary, secondary, and mature oocytes (Figures 4A, B). In the ${\mathrm{dnd}1}^{+/-}$ (Aa) female individuals, there seemed to be no difference from the ${\mathrm{dnd}1}^{+/+}$ (AA) heterozygote females, which maintained a developed ovary structure (Figures 4C, D).

In the male individual groups, we observed that the ${\mathrm{dnd}1}^{+/+}$ (AA) individuals maintained a well-developed testis structure, with spermatogonia, spermatids, and spermatozoa (Figures 4E, F). Similarly, the ${\mathrm{dnd}1}^{+/-}$ (Aa) male individuals had completely developed testes, which were not different from those of the ${\mathrm{dnd}1}^{+/+}$ (AA) (Figures 4G, H). The all-male phenomenon was observed in the ${\mathrm{dnd}1}^{-/-}$ (aa) individuals, compared with the ${\mathrm{dnd}1}^{+/+}$ (AA) and ${\mathrm{dnd}1}^{+/-}$ (Aa) individuals, and in the mutant of the homozygous knockout mutant (aa) groups, the ${\mathrm{dnd}1}^{-/-}$ (aa) zebrafish completely lost their germ cells, and the left space of the tube-like testes was occupied by adipocytes (Figures 4I, J).

**FIGURE 5:**
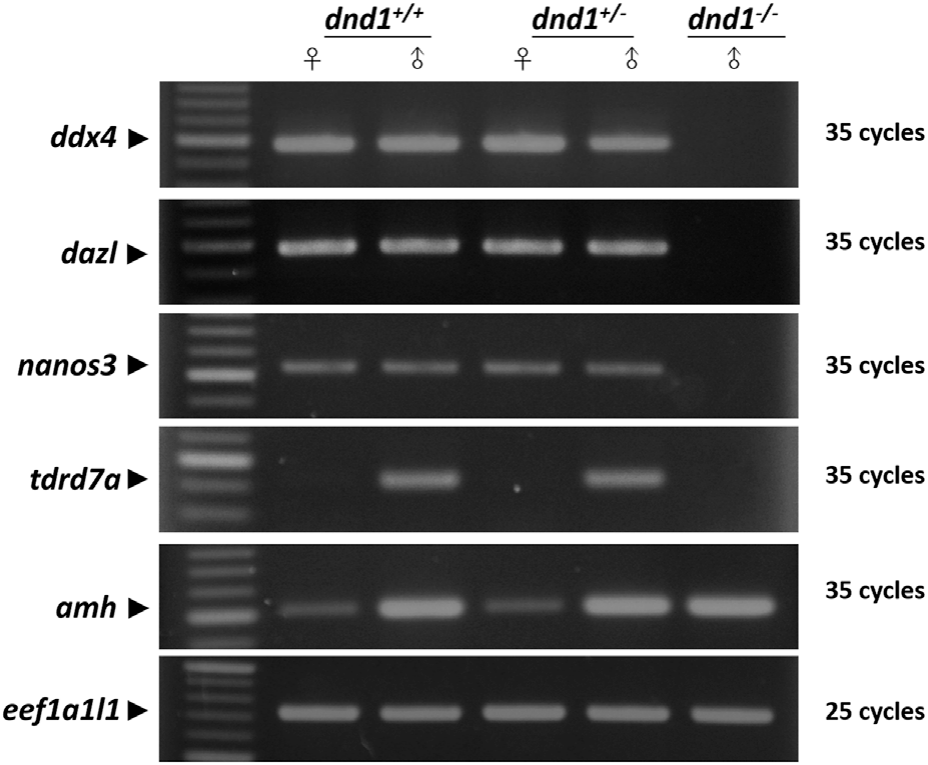
Analysis of the expression of germ-cell-related genes between the wild-type ( $\left.{\mathrm{dnd}1}^{+/+}\right)$ , heterozygous ${\left(\mathrm{dnd}1\right.}^{+/-}),$ and homozygous $\left({\mathrm{dnd}1}^{-/-}\right)$ mutant zebrafish through semi-quantitative RT-PCR.

To determine whether the ${\mathrm{dnd}1}^{+/+}$ (AA), ${\;dnd1}^{+/-}$ (Aa), and ${\mathrm{dnd}1}^{-/-}$ (aa) knockouts would affect the expression levels of the germline-related genes *ddx4, dazl, nanos3,* and *tdrd7a,* the gonadal tissue of the testes and ovaries from ${\mathrm{dnd}1}^{+/+}$ (AA) and ${\mathrm{dnd}1}^{+/-}$ (Aa) and tube-like gonads from ${\mathrm{dnd}1}^{-/-}$ (aa) were sampled for RNA extraction for analysis through semi-quantitative RT-PCR. In this experiment, the *anti-mullerian hormone* (*amh*) was used as a positive control, and *eef1α1l1* was the stable reference gene. In both the ${\text{dnd}1}^{+/+}$ (AA) and ${\mathrm{dnd}1}^{+/-}$ (Aa) female and male groups, *ddx4, dazl,* and *nanos3* had similar expression levels, but in ${\mathrm{dnd}1}^{-/-}$ (aa), the germ-cell-related gene expression was found to be lacking (Figure 5). While ${\mathrm{dnd}1}^{+/+}$ (AA) and ${\mathrm{dnd}1}^{+/-}$ (Aa) showed a specific expression of *tdrd7a* in the testes, ${\mathrm{dnd}1}^{-/-}$ (aa) also showed a lack of expression (Figure 5). The gene *amh* showed a positive expression in both sexes of ${\mathrm{dnd}1}^{+/+}$ (AA), ${\mathrm{dnd}1}^{+/-}$ (Aa), and ${\mathrm{dnd}1}^{-/-}$ (aa) (Figure 5). As *amh* is expressed in somatic cells of yolk-forming oocytes and spermatogonia gonad tissue, in the three groups of genotypes, males had a higher expression level than the females, but no difference was observed in *amh* expression between the same sexes for the three genotypes (Figure 5) by quantitation of *amh* bands with ImageJ analysis and normalized with *eef1α1l*. The *amh* in ${\mathrm{dnd}1}^{-/-}$ (aa) showed comparable expression level with male fish of ${\mathrm{dnd}1}^{+/+}$ (AA), and ${\mathrm{dnd}1}^{+/-}$ (Aa). This indicates that the germ-cell-specific gene expression of the ${\mathrm{dnd}1}^{-/-}$ (aa) individual was lost, but it also showed that gene expression in the gonad somatic cells was maintained.

#### 3.2.3 Sexual characteristics and courtship behavior analysis

**FIGURE 6:**
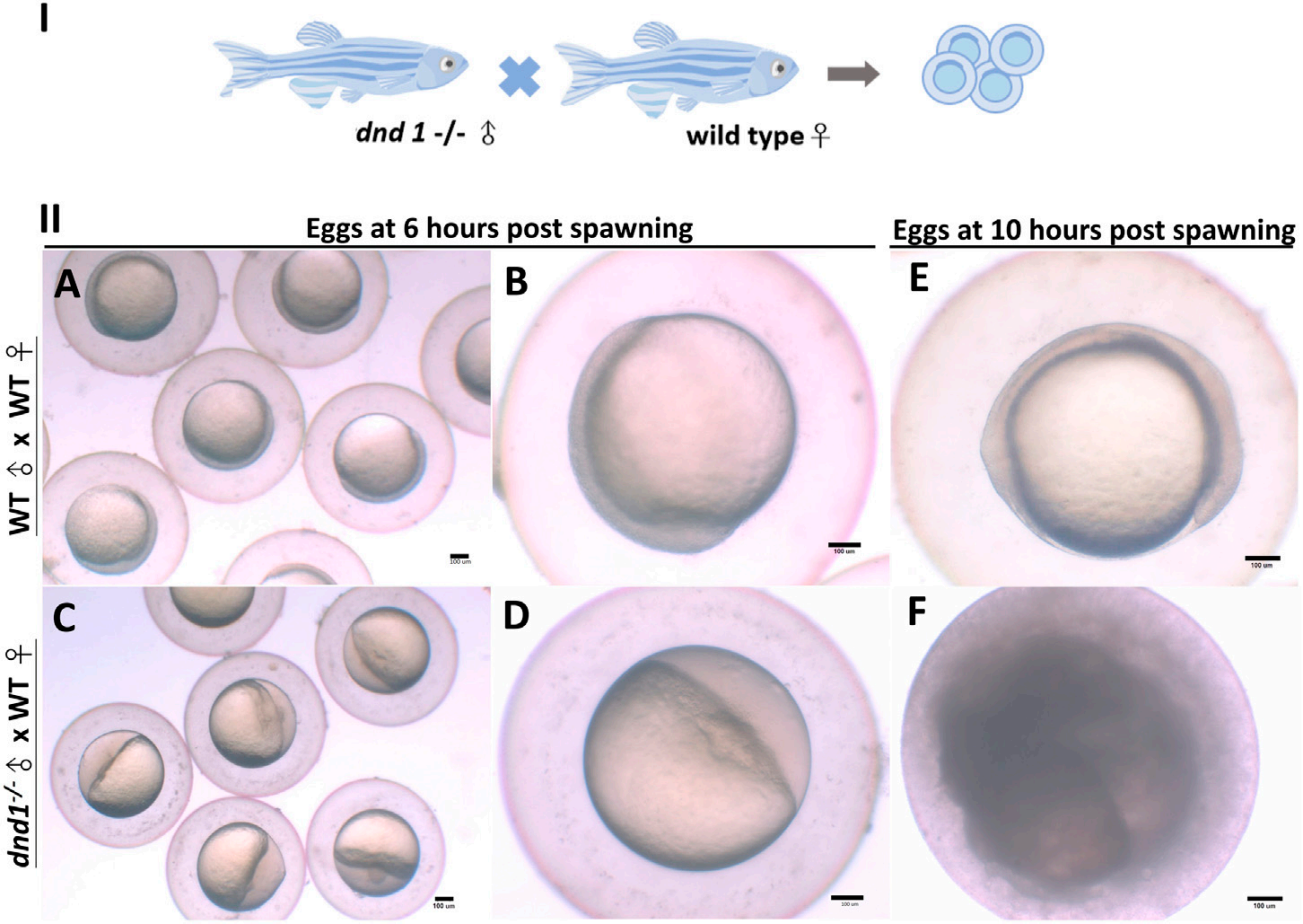
**(I)** *dnd1* ^*−/−*^ zebrafish were all male, with a normal courtship behavior. **(II)** Spawned eggs from WT female mating with *dnd1* ^*−/−*^ male and wild-type zebrafish. **(A)-(B)** Embryos spawned by wild-type zebrafish after 6 h and **(E)** after 10 h. **(C)**-**(D)** Eggs spawned by mutant male zebrafish and wild-type female zebrafish after 6 h and **(F)** after 10 h. Bars = 100 μm.

We analyzed the mating behavior and fertilization capabilities of the *dnd1*-knockout zebrafish. The results show that ${\mathrm{dnd}1}^{-/-}$ (aa) are male and sustain a normal courtship behavior, which allows the wild-type female zebrafish to spawn eggs (Figure 6I). The spawned eggs were collected and observed under a dissecting microscope. Normally, fertilized zebrafish embryos reach a 50% epiboly stage after 6 h (Figures 6IIA, B). The eggs that are spawned by ${\mathrm{dnd}1}^{-/-}$ (aa) and wild-type female zebrafish show half of the animal pole and half of the vegetal pole in terms of their shape. This is a phenomenon that indicates that eggs are not fertilized (Figures 6IIC, D). Normally, fertilized embryos achieve a 90% epiboly stage after 10 h (Figures 6IIE). The spawned eggs of the ${\mathrm{dnd}1}^{-/-}$ (aa) and wild-type female zebrafish showed cell death (Figures 6IIF).

To confirm the results reported above, we sampled two ${\mathrm{dnd}1}^{-/-}$ (aa) individuals and randomly mated them with wild-type female zebrafish, and the survival rates of the spawned eggs were recorded. Additionally, we randomly selected female and male wild-type groups as the controls. In the wild-type groups, 136, 97, 122, and 178 eggs were spawned through mating, and the survival rates of the eggs averaged over 90% after 24 h (Supplementary Figure S1). In the groups of ${\mathrm{dnd}1}^{-/-}$ (aa) (Gordon, 1997), 120, 202, 96, and 113 eggs were collected. In the groups of ${\mathrm{dnd}1}^{-/-}$ (aa) (Ozato et al., 1992), 184, 111, 89, and 117 eggs were spawned by wild-type female zebrafish, and the eggs were unfertilized and then underwent cell death after 24 h in both the ${\mathrm{dnd}1}^{-/-}$ (aa) (Gordon, 1997) and ${\mathrm{dnd}1}^{-/-}$ (aa) (Ozato et al., 1992) groups (Supplementary Figure S1). This result showed that the *dnd1-*knockout zebrafish were infertile but had normal sexual characteristics.

### 3.3 Infertility control applied to fluorescent zebrafish

**FIGURE 7:**
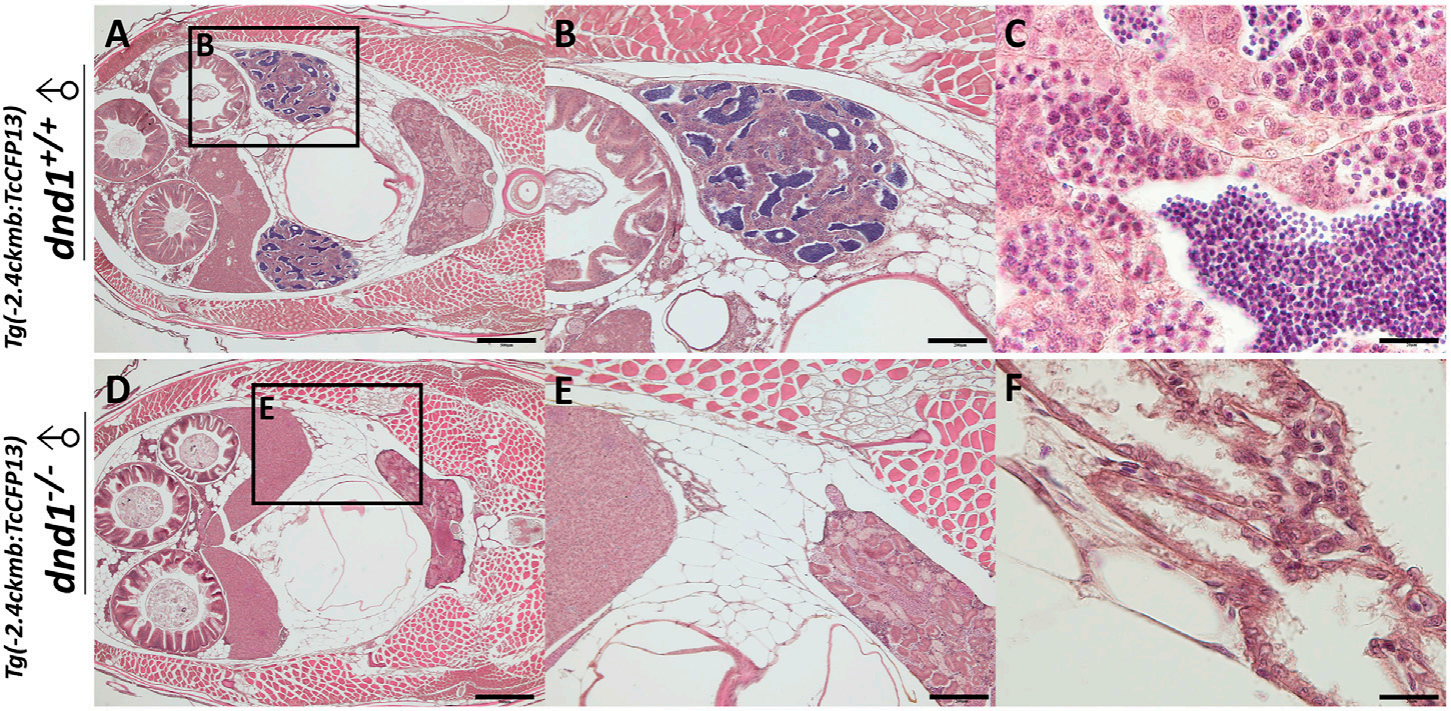
Histological analyses of fluorescent zebrafish testis structure. **(A)-(C)** *dnd1^+/+^* fluorescent zebrafish. **(D)-(F)** *dnd1*-knockout homozygote fluorescent zebrafish. Bars = 500 μm **(A, D)**, 200 μm **(B, E)**, and 20 μm **(C, F)**.

According to the results of the experiments above, we used wild-type zebrafish as a model to reveal the targeted mutagenesis in zebrafish *dnd1*, which may disrupt the germ cell formation, leading to a loss of germ cells and the ${\mathrm{dnd}1}^{-/-}$ (aa) individual losing the ability to fertilize eggs. Furthermore, we applied the infertility control to fluorescent zebrafish to explore the practicalities of the infertility control technology. We analyzed the fluorescent zebrafish with the *dnd1* mutant strain by mating the cyan fluorescent zebrafish *Tg(-2.4ckmb:TcCFP13)* with the heterozygotes of pattern D (−7/WT) to generate the $F_{1}$ generation. Genotyping was conducted to select the heterozygotes of the individuals carrying both the heritable knockout pattern and the fluorescent pattern, inbreeding the selected individuals to generate the $F_{2}$ generation. Based on the results of our previous experiments, we focused on the analysis of the germ cell development and the egg fertilization ability of the mature individuals of the fluorescent ${\mathrm{dnd}1}^{-/-}$ (aa) zebrafish. We performed histological analysis on both cyan fluorescent ${\mathrm{dnd}1}^{+/+}$ (AA) male zebrafish and cyan fluorescent ${\mathrm{dnd}1}^{-/-}$ (aa) zebrafish after genotyping. A completely developed testis structure was observed in the cyan fluorescent ${\mathrm{dnd}1}^{+/+}$ (AA) candidates, in which testes developed on both sides of the body (Figures 7A–C). Moreover, in the cyan fluorescent ${\mathrm{dnd}1}^{-/-}$ (aa) zebrafish, we observed the tissue sectioning, showing no testis structure, a loss of spermatozoa, or even primary-stage germ cells in the fluorescent *dnd1-*knockout individuals (Figures 7D–F).

**FIGURE 8:**
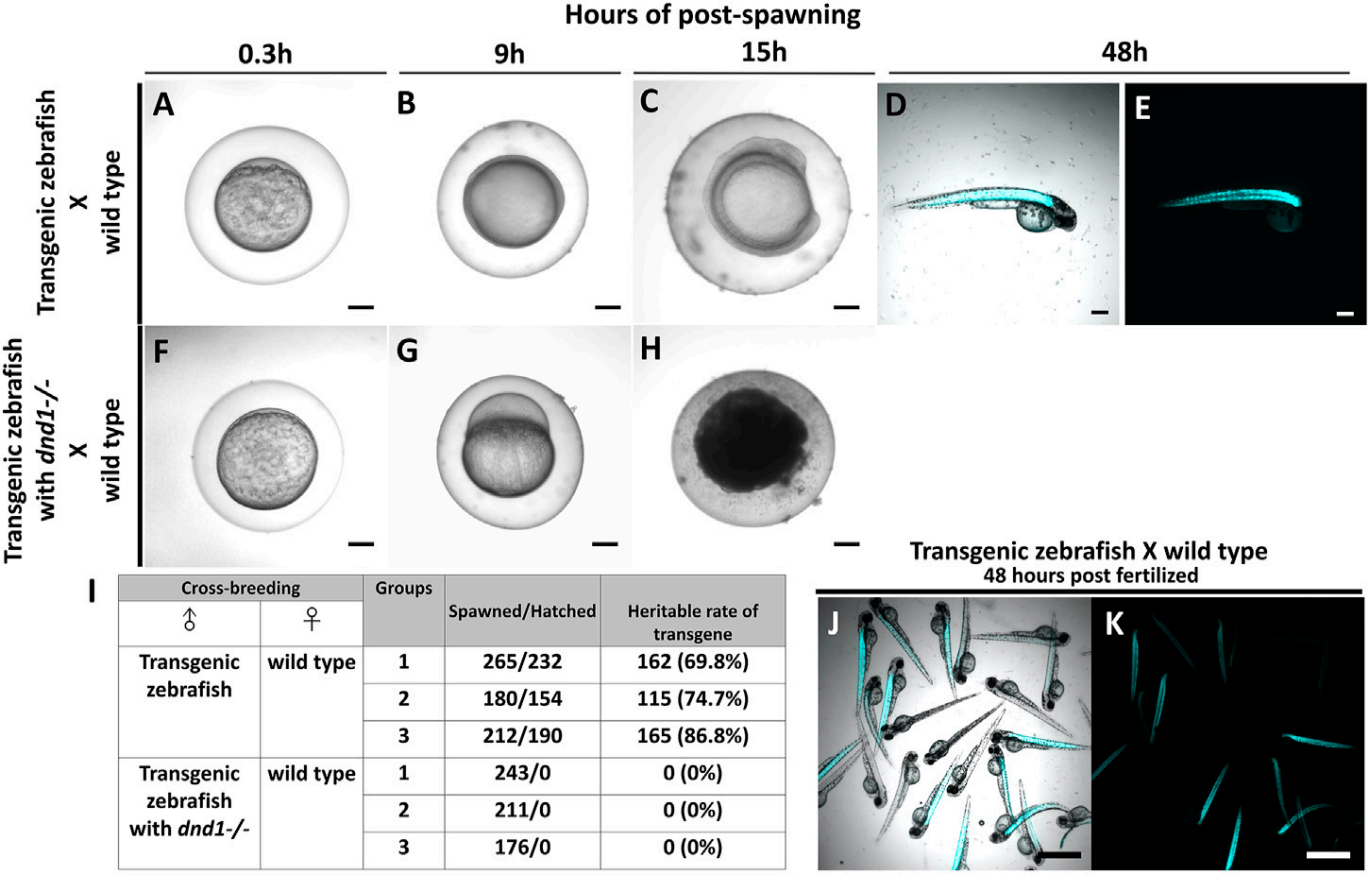
Transgenic zebrafish with *dnd1* mutant can effectively avoid the transgene inherited. The eggs spawned by transgenic zebrafish and wild type zebrafish **(A)** .3 h **(B)** 9 h **(C)** 15 h **(D)** 48 h of post spawning under light view **(E)** 48 h of post spawning under dark view. The eggs spawned by transgenic zebrafish with *dnd1* mutant and wild type zebrafish **(F)** .3 h **(G)** 9 h **(H)** 15 h. **(I)** The heritable rate counting of transgene in cross-breeding group. Top view of hatched offspring of cross-breeding by transgenic zebrafish and wild type zebrafish. **(J)** light view **(K)** dark view. Bars = 200 μm **(A–H)** and 500 μm **(J, K)**.

In case, the zebrafish which carry transgenes go into the river and mate with the wild type zebrafish, it causes widespread of the transgene as the transgenes are heritable. Therefore, we conduct the breeding experiment to simulate the escapement of the transgenic zebrafish, and to study the gene flow of the foreign gene. At the same time, we test whether the transgenes are completely inherited after performing *dnd1* mutation treatment. For easy observation, we selected the strain with muscle florescent expression to perform. Both transgenic zebrafish, transgenic zebrafish with ${\mathrm{dnd}1}^{-/-}$ (aa), and wild type zebrafish are selected randomly for the breeding experiment. We set the breeding experiment candidate ratio to 1:1 in all groups and repeated it three times, all the eggs were collected after spawned (Figures 8A, F). We found the embryos developed normally and reached the bud stage in 9 h of post-spawning in the cross-breeding groups of transgenic zebrafish and wild type zebrafish (Figure 8B). Conversely, we observed a lack of development in the eggs that spawned by wild type females in the cross-breeding groups of transgenic zebrafish with ${\mathrm{dnd}1}^{-/-}$ (aa) and wild type zebrafish (Figure 8G). The embryos spawned by the transgenic zebrafish and wild type zebrafish continuously developed to the somite stage after 15 h post-spawning (Figure 8C); however, the eggs that were spawned by the transgenic zebrafish with ${\mathrm{dnd}1}^{-/-}$ (aa) and wild type zebrafish were all dead (Figure 8H). In this experiment, we found that transgenic zebrafish successfully mate with wild type zebrafish, the eggs are fertilized and develop normally during embryogenesis. Continuously, after 48 h post spawned, we found the zebrafish hatched, also some of the individuals had observed the fluorescent signal (Figures 8D, E). Conversely, in the transgenic zebrafish with the *dnd1* mutant group, the wild type female zebrafish mated with transgenic zebrafish normally. But we found the eggs were unfertilized, and show abnormal development, and undergo death within 15 h post spawning.

Further, we calculated the total number of eggs that were spawned in each cross-breeding group, and also verified the heritable rate of transgenes. In the cross-breeding groups of transgenic zebrafish and wild type zebrafish, we collected 265, 180, and 212 spawned eggs, a total of 232, 154, and 190 eggs were hatched (Figure 8I). We observed the fluorescent signal under the microscope to calculate the heritable rate of the transgene, we found the transgenic zebrafish may have 69.8%, 74.7% and 86.8% of heritage (Figures 8I–K). In the cross-breeding groups of transgenic zebrafish with *dnd1* mutant and wild type zebrafish, a total of 243, 211, and 176 eggs had collected, but all the eggs were found unfertilized (Figure 8I). From the result, the transgenic zebrafish are infertile after *dnd1* mutant, and success to avoid the transgene hereditary.

Finally, we performed a larger scale of breeding experiment to confirm the infertility effect of transgenic fish with ${\mathrm{dnd}1}^{-/-}$ (aa), with a total of 40 transgenic fish with ${\mathrm{dnd}1}^{-/-}$ (aa) zebrafish selected randomly (Supplementary Figure S2). We set the breeding experiment candidate ratio of transgenic cyan fluorescent zebrafish with ${\mathrm{dnd}1}^{-/-}$ (aa) knockout and wild type female zebrafish to 1:1 (Supplementary Figure S2A). The *dnd1* homozygous mutant male fluorescent zebrafish has normal courtship behavior with wild-type female to spawning eggs (Supplementary movie). Ten transgenic fish with ${\mathrm{dnd}1}^{-/-}$ (aa) zebrafish were examined in each experiment, and the breeding experiments were repeated four times to collect data on 40 pairs of zebrafish. Simultaneously, we used 10 pairs of wild type zebrafish as the control groups. The eggs were collected after mating, then we calculated the total number of eggs that were spawned by the wild type inbreeding groups and the groups of transgenic zebrafish with cyan fluorescent that mated with the wild type females (Supplementary Figures S2B, C, E, F). From the four breeding experiments, 1 h post-spawning in the wild-type inbreeding groups, we collected 1,347, 1,209, 1,432, and 1,156 eggs from 10 pairs of zebrafish, with a 100% survival rate (Supplementary Figure S2D); in the transgenic zebrafish with ${\mathrm{dnd}1}^{-/-}$ (aa) zebrafish groups, we collected 1,530, 1,236, 1,282, and 1,498 eggs, with a 100% survival rate from 10 pairs of zebrafish that mated with the transgenic zebrafish with ${\mathrm{dnd}1}^{-/-}$ (aa) zebrafish (Supplementary Figure S2G). At 6 h post-spawning, we recorded the survival status of two groups, in which no significant death was observed (Supplementary Figures S2B, E). At 24 h post-spawning, the survival rate of the four control groups was 95% or higher, with no significant number of dead eggs observed (Supplementary Figures S2C, D); in the transgenic zebrafish with ${\mathrm{dnd}1}^{-/-}$ (aa) zebrafish groups, we found that all of the eggs were dead (Supplementary Figures S2F, G), unlike the control. The result above shows that the infertility control technology could actually be applied to prevent the gene flow of transgene because the mutant *dnd1* individuals lost germ cells and the ability to fertilize eggs.

## 4 Discussion

As the demand for fishery resources has increased due to global population growth, marine fisheries are gradually starting to face a lack of resources. To reduce the threats relating to overfishing, to protect marine ecology, the supply of aquatic products has gradually shifted toward aquaculture (Tidwell and Allan, 2001). However, the demand for land resources and feed supplies has restrained the growth of the aquaculture industry (Cao et al., 2015). Therefore, nowadays, in the aquaculture industry, optimizing the growth, nutritional value, and disease resistance of farmed fish species, shortening their breeding times, and maximizing the production values are the main goals of development (Tidwell and Allan, 2001). To improve the quality of breeding livestock, selective breeding is commonly used in the industry. In addition, importing exotic species to adapt to cultural conditions is usually applied in the aquatic industry through global trade (Perez et al., 2003). This may increase cultivation and bring several advantages in terms of development (Gjedrem et al., 2012). However, if the artificially modified species escape from our cultural environment, the biodiversity and abundance of native species may be affected because the performance and adaptability of the selected individuals may be better than those of the native species (Lind et al., 2012).

According to previous studies, transgenic technology is a technique that could be applied in the aquatic industry for both ornamental fish and food fish, inserting exotic genes to provide the target species with the greatest performance, thus creating huge business opportunities (Gong et al., 2003; Clausen and Longo, 2012). Unfortunately, if the genetically modified species established using the transgenic technique that carry the exotic gene from the other species escape from our cultivation area, the potential risks posed to our ecosystem cannot be assessed. Thus, the application of transgenic technology has been regulated under GMO regulations. Based on the situation mentioned above, culturing sterile fish may allow the ecological risks to be avoided whenever an unexpected out-flow happens. We believe that applying effective and practical infertility control technologies is important.

DND1 is a vertebrate RNA-binding protein that was first discovered in zebrafish (Weidinger et al., 2003; Kedde et al., 2007b). Previous studies have shown that the amino acid sequence of DND1 contains six functional regions, including the N-terminal region (NR), the RNA-recognition motif (RRM), and four C-terminal regions (CR 1–4) (Slanchev et al., 2009). The RRM is the most important functional region in DND1 and helps to transfer DND1 from the nucleus to the germ cell granules. While CR has ATPase activity, required for the development of PGCs and involved in the mRNA protection of downstream genes, such as *nanos3* and *tdrd7a*, mutations in this region will reduce the number of PGCs (Liu and Collodi, 2010). In previous research, the knockdown of *dnd1* expression using a morpholino in zebrafish, *Xenopus*, medaka, Atlantic cod, and other species resulted in a loss of germ cells (Weidinger et al., 2003; Youngren et al., 2005; Horvay et al., 2006; Goto et al., 2012; Northrup et al., 2012; Skugor et al., 2014; Su et al., 2014; Linhartova et al., 2015; Su et al., 2015; Hong et al., 2016). In zebrafish, the knockdown of *dnd1* resulted in a loss of germ cells due to the mis-migration and transdifferentiation of PGCs, which failed to protect the fate of the cells (Gross-Thebing et al., 2017). As *dnd1* is a critical gene that has been proven to be important in the migration and cell fate stability of PGCs, several researchers have focused on *dnd1* to perform gene disruption to explore the possibility of infertility control. In 2003, infertility control was achieved by the knockdown of the *dnd1* gene’s expression using a morpholino, but performing a microinjection of one-cell fertilized eggs is an operation that is too technically complex to be applied in the industry (Weidinger et al., 2003). In 2015, researchers established the immersive bath method of Vivo-Morpholino-*dnd1* and proposed the knockdown of *dnd1*. This technique is indeed simpler to operate than microinjection, but it may be too difficult to apply in industrial applications, as the Vivo-morpholino treatment is quite expensive and non-inheritable (Wong and Zohar, 2015a). The different conditions in terms of the various fish species and egg sizes may hinder the effectiveness of the technique, and it may therefore not be able to guarantee consistency in achieving complete infertility.

In this study, gene editing was performed using CRISPR/Cas9, and the target mutagenesis site for gRNA was located before the translation of functional regions. According to the results of this study, the in-frame stop codon generated by CRISPR/Cas9 showed an abnormal translation, resulting in the *dnd1* gene showing a loss of function. This technology applied in model species, such as zebrafish, could help us to explore whether the lack of *dnd1* generated by CRISPR/Cas9 could allow complete infertility to be achieved and a complete analytical procedure to be established in a shorter period of time. We found a loss of fertilization ability in both wild-type and fluorescent zebrafish, and the targeted mutagenesis of *dnd1* using the CRISPR/Cas9 system resulted in a loss of germ cells in homozygotic individuals, which is consistent with the results presented above.

In previous studies, it was proved that *nanos3* and *tdrd7a* were protected by DND1, which competed with the binding site of the 3′ untranslated region (UTR) of the gene’s mRNA to hinder its microRNA-induced degradation (Kedde et al., 2007a; Aguero et al., 2017). We analyzed *dnd1*-related genes, such as *nanos3*, *tdrd7a*, and *ddx4*, in *dnd1*-knockout individuals. In *dnd1*-knockout homozygotic individuals, germ-cell-related genes resulted in a lack of expression, but there was a gonadal somatic cell expression of the *amh* gene. We conjecture that the knockout of *dnd1* caused a loss of germ cells, but with no significant effect on the gonadal somatic cells. According to research on *dnd1* knockdown in medaka, injecting different concentrations of a morpholino leads to a direct effect on the number of PGCs (Hong et al., 2016). However, we did not observe germ-cell-related gene downregulation or histological evidence in heterozygotic individuals. We could only determine whether there were differences in the gene expression levels and the fertility capacity to determine whether there was a significant difference between the heterozygotes and wild types.

**FIGURE 9:**
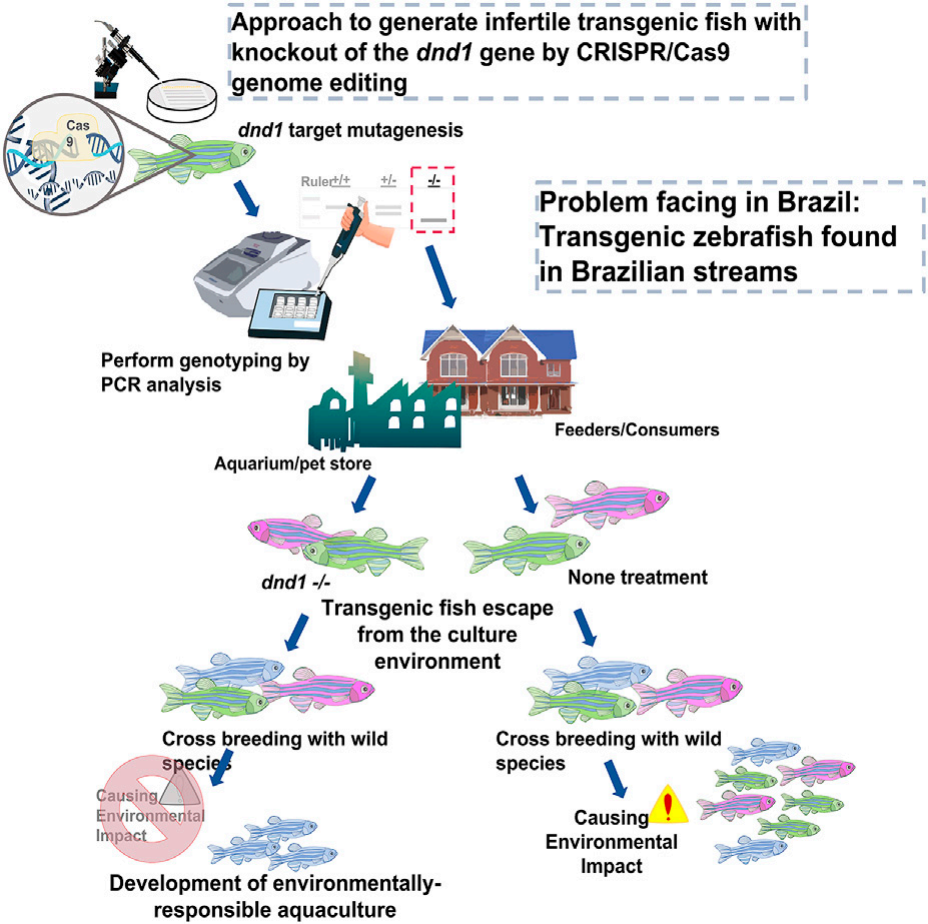
Approach to generate infertile transgenic fluorescent zebrafish with knockout out of the *dnd1* gene by CRISPR/Cas9 genome editing. We demonstrate that the *dnd1* knockout by CRISPR/Cas9 genome editing could be applied to the infertility control of transgenic fluorescent zebrafish to prevent them from cross-breeding with wild species when they escape into the wild field. It can promote the development of an environmentally responsible aquaculture.

In this study, we found that the all-male phenotype in *dnd1*-knockout zebrafish by CRISPR/Cas9 was complete sterile, although the males still maintained normal courtship behavior. In the study of *dnd1*-knockdown zebrafish, we observed the same all-male phenomenon (Pradhan and Olsson, 2018). According to previous research, a lack of germ cells in zebrafish, medaka, and tilapia leads to their sex determination in the all-male phenotype, as the number of germ cells may affect the differentiation mechanism (Slanchev et al., 2005; Kurokawa et al., 2007; Li et al., 2014). In our study, CRISPR/Cas9 technology, which needs only prepare a single guide RNA and Cas9 mRNA to induce dsDNA break for genome editing, was used to knockout *dnd1* gene for complete infertility control of transgenic fish. The CRISPR/Cas9 genome editing technology is a more easy, cheap and precise way than ZFNs and TALENs. As zebrafish as a model species, to establish an integrated infertility control procedure by CRISPR/Cas9 genome editing from the development to the analysis further is necessary. Also, this study mainly focused on the application of infertility control on transgenic fish, and we provided evidence to emphasize the practicality of this technique. We established *dnd1*-knockout zebrafish using CRISPR/Cas9 gene editing technology and achieved complete infertility in homozygotic individuals of transgenic cyan fluorescent zebrafish. While this application of preparatory work to perform gene editing is complicated, as long as heterozygous mutants are established, and the stable heritable knockout genome sequences are selected, followed by the interbreeding of heterozygotes, homozygotic individuals can be analyzed by fin cleaving, PCR amplification and genotyping (Figure 9). Besides the generation of completely infertile fish, infertility species may be used as germ cell transplantation carriers to produce the germ cells of different species (Saito et al., 2008; Yazawa et al., 2010; Yazawa et al., 2013; Pacchiarini et al., 2014; Morita et al., 2015; Lujic et al., 2018). However, the idea of infertility control by knockout strategy seems to be impractical when it is put into the application of in aquaculture scale. As the verification of genotype may require PCR amplification procedure and the inheritable homozygote ratio which is around 1/4 of the total offspring. As fertile *dnd1* heterozygous mutants still can breed, they have to be kept in breeding company under strict regulation to prevent from escape. Only sterile transgenic fluorescent zebrafish with *dnd1* homozygous mutations can be sold to local aquariums and consumers. There is no practical application if fertility cannot be restored in *dnd1* homozygous mutants for mass production of sterile offspring. In Atlantic salmon, rescue of germ cells in *dnd1* knockout embryos by CRISPR/Cas9 opened possibility to produce inherited sterility (Guralp et al., 2020). Development of practical mass production technology of sterile fish still need to be conquered by more studies and efforts. Nevertheless, practicality and stability are the most important elements in the infertility control technique. We propose that this technique may be suitable for the selective breeding of fish species to achieve a higher product value or to achieve transgenic individuals, which infertility control must be applied to ensure that the transgenic individuals are completely sterile.

Nowadays, the development of biotechnology is mature, and many important breeding fish species have developed breeding technologies with great application potential. In addition to being applied to the industry, these technologies must also pass numerous regulatory and analytical tests. Especially for the artificial selective breeding or genetic modified fish, the most concern of us is to protect our natural primitive ecology by avoiding the foreign gene or dominant species from invading or affecting the wild population, whereas the application of infertility control technique is one of the keys. In this study, we introduce a complete procedure from the development of *dnd1* knockout candidates to the analyses of stable heritable rates of homozygotes zebrafish, with multiple breeding experiments to confirm the sterile fish which did not maintain their reproductive ability. It is important to develop and employ effective infertility control in industry practice, as this may allow us to protect our ecosystem and build up a responsible aquaculture environment. Although our study achieved infertility control by genome editing of the *dead end* gene, which has been reported in zebrafish by ZFNs, we still believed that using new gene editing technology ‘CRISPR/Cas9′ may help the progress of infertility control applicated in the industry, as the *dead end* gene is conserved between vertebrates and the accomplishment of the target mutagenesis is more simple, faster and precise. Zebrafish is the most important model species, and the completeness and systematic process description are vitally important for the reference of other species.

## 5 Conclusion

To avoid the ecological risks associated with escaping transgenic fish into the aquatic environment, the development of an effective sterilization strategy is essential. *Dead end* (*dnd1*) is a critical gene that plays an essential role in the migration, survival, and cell fate maintenance of primordial germ cells (PGCs). In this study, we report the targeted mutagenesis of the *dnd1* gene in zebrafish by using CRISPR/Cas9 genome editing technology to achieve complete sterility in *dnd1*-gene-knockout non-transgenic zebrafish and transgenic fluorescent zebrafish, as the model for the infertility control in farmed ornamental fish and transgenic fish, especially in transgenic fluorescent ornamental fish.

## Data Availability

The datasets presented in this study can be found in online repositories. The names of the repository/repositories and accession number(s) can be found in the article/Supplementary Material.
