# Supplementary material for: Infertility control of transgenic fluorescent zebrafish with targeted mutagenesis of the dnd1 gene by CRISPR/Cas9 genome editing
Source: Front Genet. 2023 Jan 13;14:1029200. doi: 10.3389/fgene.2023.1029200 (PMC9881232; doi:10.3389/fgene.2023.1029200)
Supplement: Supplementary file 2 [file Table1.DOCX]

Supplementary Material

## Supplementary Figures

**
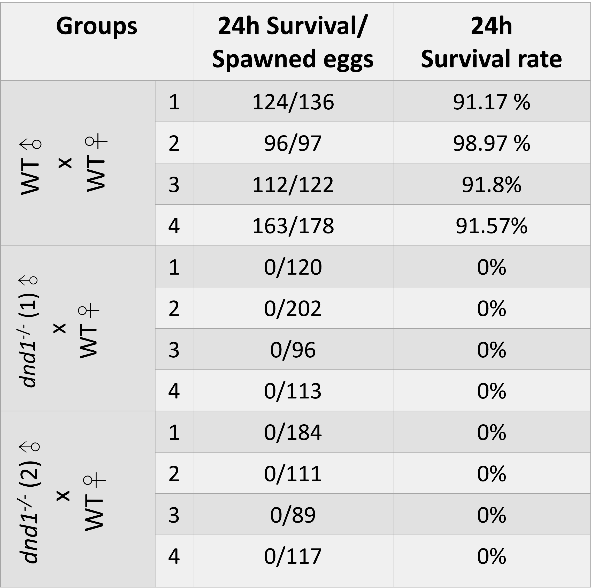

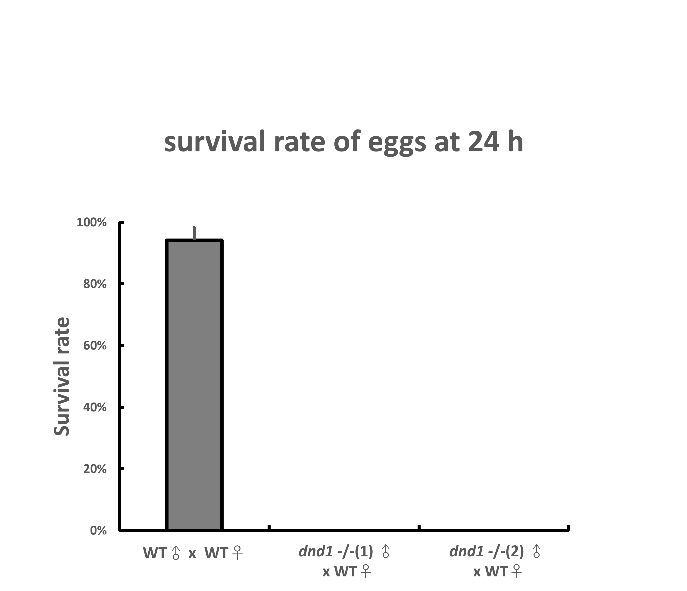
**

**Supplementary Figure 1.** The survival rate for eggs spawned by wild-type female zebrafish mating *dnd1* mutant male zebrafish compared with mating wild-type male zebrafish.


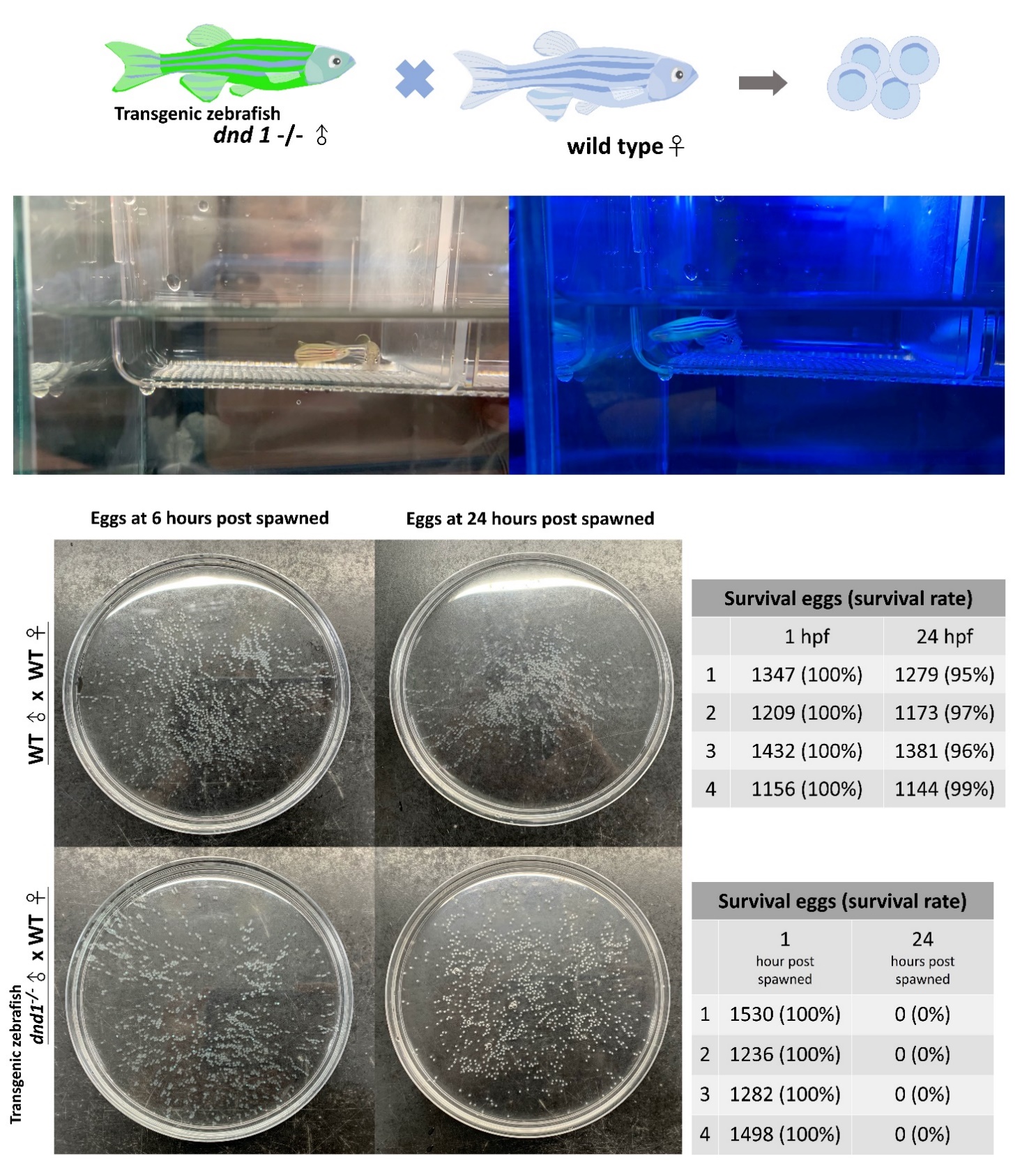


**A**

**B**

**C**

**D**

**E**

**F**

**G**

**Supplementary Figure 2.** Fluorescent *dnd1^-/-^* zebrafish with a normal courtship behavior and differences between the eggs spawned by fluorescent *dnd1*-mutant zebrafish and wild-type zebrafish. **(A)** Schematic diagram of fluorescent *dnd1^-/-^* zebrafish courtship behavior in light view and dark view. **(B)** Eggs spawned by 10 pairs of wild-type zebrafish after 1 hour and **(C)** 24 hours. **(D)** Fertilization ability of wild-type zebrafish. **(E)** Eggs spawned by fluorescent *dnd1-*mutant male zebrafish and wild-type female zebrafish after 1 hour and **(F)** 24 hours. **(G)** Fertilization ability of fluorescent *dnd1-*mutant male zebrafish.

**Supplementary Movie**

**
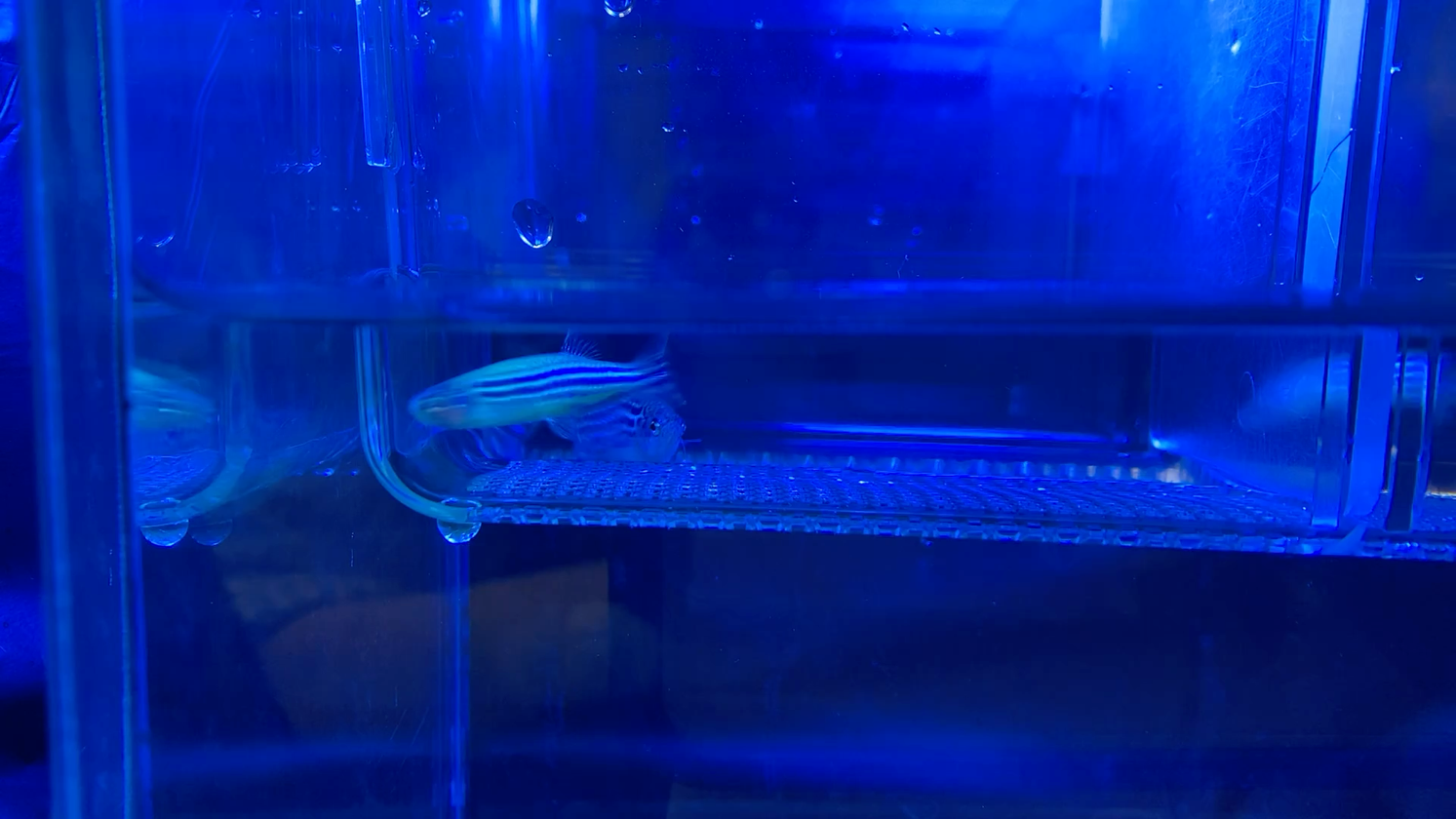
**The *dnd1* homozygous mutant male cyan fluorescent zebrafish has normal courtship behavior with wild-type female to spawning eggs (double click the picture below to see the movie)
